# Perceptions of recovery and rehabilitation in people with brain injury in Spain. A qualitative study

**DOI:** 10.1111/hex.13471

**Published:** 2022-03-14

**Authors:** Sagrario Pérez‐de la Cruz

**Affiliations:** ^1^ Department of Nursing, Physical Therapy, and Medicine University of Almería Almería Spain

**Keywords:** acquired brain injury, perception, physiotherapy, qualitative descriptive, rehabilitation, Spain

## Abstract

**Introduction:**

Acquired brain injury (ABI) is a condition that severely impairs the personal, family, social and professional lives of the individuals who experience it. The aim of this study was to gain insight into ABI patients' perceptions of their condition and rehabilitation process so that physiotherapists can approach their treatment in a more comprehensive, satisfactory manner.

**Methods:**

A qualitative study was carried out with individual interviews, and focus group sessions (semi‐structured interviews) were held with 33 individuals from various associations.

**Results:**

Four themes emerged in this study: physiotherapy treatment, changes in lifestyle, patients' feelings about their condition and aspirations for the future. The participants reported that their condition had led to multiple changes in their personal and family lives that were not always positive.

**Conclusions:**

These findings may be useful for identifying ways to increase acceptance of their condition and design a comprehensive rehabilitation programme for these patients and their families. The psychosocial needs of ABI patients could be optimized by providing good physical care through effective communication within the rehabilitation environment where communication between professionals and patients prevails, to meet their real needs and expectations.

**Patient or Public Contribution:**

This study was conducted based on interviews with adult ABI patients regarding their experiences concerning their rehabilitation process and daily life.

## 1 INTRODUCTION

Acquired brain injury (ABI) is brain damage that occurs after birth due to vascular and/or traumatic reasons. It is a major health problem and one of the main causes of disability.^1^ ABI is an umbrella term encompassing many aetiologies including vascular (stroke) and traumatic causes.^2^

Data from the Spanish National Institute of Statistics suggest that in Spain, there are approximately 500,000 individuals with ABI. Of these, 78% are caused by stroke, which is the main cause of ABI.^3^ The second most frequent cause is traumatic brain injury (TBI). It is estimated that there are between 50,000 and 75,000 individuals living in these circumstances in Spain and another 2500 in vegetative or minimally conscious states.^4^ The most severe cases are found in very young, male patients (under 30 years of age) with a mean life expectancy of around 35 years. Almost all of them are of working age or about to finish higher education.

The large number of cases and the ongoing progress in medicine and technology have led to increased survival rates among individuals with severe brain injury. Increased survival rates have led to an increase in the number of sequelae affecting not only individuals with ABI but also their families and their social and work environments, considerably impacting their quality of life.^3^ Most survivors experience sequelae affecting sensorimotor, cognitive, emotional and/or behavioural aspects with a direct impact on their performance of activities of daily living (ADLs).^5^, ^6^ Their participation in ADLs is often disrupted to the point of abandoning these activities or becoming completely dependent on others to perform them. For this reason, one of the primary goals of rehabilitation is to achieve as much independence and functionality as possible.^7^ Another key objective is to maximize patients' social engagement to improve their well‐being and minimize the burden and distress for their families.^8^

Rehabilitation professionals should be aware of the different aspects related to the evolution of those with ABI and their recovery process when developing proposals for joint action between different professionals involved, adapting to the individual, family, work and social needs of patients.

Despite the growing number of trials and systematic reviews assessing the effectiveness of rehabilitation,^9^, ^10^, ^11^, ^12^ the views and experiences of ABI patients regarding their rehabilitation process have received little attention in the literature. A recent study by Pindus et al.^13^ on the perceptions of patients and their caregivers concluded that they felt neglected by public services and found it very difficult to resume their previous activities. Further qualitative studies are needed to properly understand the effects of therapies on patients from their own perspectives.

In this sense, qualitative research can provide valuable insights into the complexity of harmonizing therapeutic criteria and inform the design of suitable interventions. Researchers are increasingly encouraged to work in partnership with individuals who have experienced particular health conditions to understand the key factors influencing appropriate, tailored therapy programming and bridge the gap between clinical theory and practice.^14^

The aim of this study was to gain insight into ABI patients' perceptions of their condition and rehabilitation process so that physiotherapists can approach their treatment in a more comprehensive, satisfactory manner. In addition, awareness of the neurological rehabilitation treatments that patients have received, the changes that have taken place in their lives, their feelings about the disease and their aspirations for the future is necessary to implement comprehensive treatments that help them to achieve the maximum possible functional status and quality of life.

## 2 MATERIALS AND METHODS

### 2.1 Design

This qualitative study (hermeneutical phenomenological method) adopted an interpretative approach to data collection and analysis.^15^ Data were collected in a rigorous manner using a combination of individual interviews and focus group sessions. This method seeks to further understand people's perceptions (motivations, meanings and their world), their interactions and the culture of social groups through a comprehensive process.

When participants were unable to attend their focus group session, they were offered the possibility of an individual interview.^16^ In‐depth interviews were conducted to gain a better understanding of participants and increase their confidence, improving the quality of their contributions and opinions. Where possible, focus group sessions were held to obtain a wider range of perspectives through dynamic group interactions. The consolidated criteria for reporting qualitative research (COREQ) checklist were used to design and report this study.^17^

### 2.2 Sample/participants

Participants were ABI patients, that is, patients with TBI, haemorrhagic stroke, arteriovenous malformation or brain tumours, and were members of ABI patient associations.

The inclusion criteria were as follows: chronic patients with a long clinical course (greater than or equal to 4 years) who have experienced some type of ABI; who have required physiotherapy treatment for their functional recovery; who have actively participated in their recovery process; who can speak and understand Spanish; and who signed the informed consent form.

The exclusion criteria were as follows: long‐term patients who have experienced some form of ABI, but whose state of consciousness, cognition and/or speech precludes verbal communication.

A snowball sampling technique was used to recruit participants. A total of 33 people made up the final sample for this study (19 males and 14 females). The aetiologies of their ABI were as follows: stroke (*n* = 18), TBI (*n* = 10), congenital malformations (*n* = 3) and infections (*n* = 2). The mean age of the participants was 42.4 years (range: 18–66), and the mean duration of their condition was 7.4 years (range: 1.5–17.6). Patients were located in central Spain.

The study was approved by the University of Almería's Bioethics Committee, Spain, with reference number UALBIO2017/008, as part of research project NCT04168164.

Participants were informed about the study objectives and characteristics by the researcher and had to sign an informed consent form to be able to participate.

### 2.3 Data collection

Data collection took place between December 2019 and September 2020 in a convenient location for the participants. A number of interviews had to be conducted by telephone due to COVID‐19 restrictions. All interviews and focus group sessions were conducted in Spanish. Sessions ranged from 25 to 70 min in length and followed a semi‐structured interview format based on observations and findings from previous similar research. The interviewer had no relationship with the participants to avoid introducing bias or prejudice of any kind. With the permission of the participants, an audio‐recording system was used so that both the interviewer and an external person could take notes on the group discussion, participants' body language and group dynamics.^18^

### 2.4 Data analysis

The audio recordings of the interviews and focus groups were transcribed verbatim and personal data were anonymized. All recordings and transcripts were double‐checked for accuracy. Audio recordings were analysed using the framework method, also known as qualitative content analysis, which produced descriptive or explanatory conclusions grouped around a variety of themes. Before establishing a thematic structure, the reviewers familiarized themselves with the interviews by listening to the recordings and examining the notes taken during the sessions. The interviews were listened to randomly, and a coding system and set of codes were created for application to the study. The process of creating, reviewing and defining the themes was carried out by the lead author (S. P. C.). The final themes and subthemes were discussed and agreed upon by the lead author and various external parties via videoconference.

All participant interviews were coded using ATLAS.ti 7.1 software. The codes were grouped into themes. The code groupings were then defined, and the data were analysed. The lead author met twice with the statistician to discuss the characteristics and differences between the different data.

## 3 RESULTS

**Figure 1:**
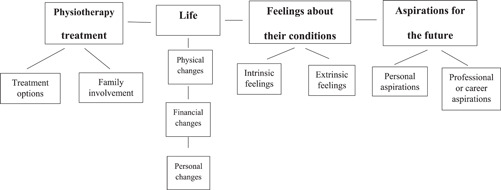
Structure of subthemes and units of meaning

Four themes emerged from the data analysis, which are shown in Table 1 and Figure 1, alongside the questions, the structure of subthemes and units of meaning (or codes).

**Table 1:** Methods

1. What was your experience of the condition?
2. What role has physiotherapy played in improving your condition since it started?
3. What role has your family played in your recovery process?
4. What physical changes occurred following your condition?
5. How do these changes affect your daily life?
6. How did your condition affect your professional life?
7. What changes occurred in your personal life?
8. How do you feel about your limitations now?
9. How do you feel about the people around you?
10. How is society treating you?
11. What would you like your personal life to be like a few years from now?
12. What are your current career aspirations?

*Note*: Interview guide including a summary of questions asked.

### 3.1 Theme 1: Physiotherapy treatment

This type of neurological patient requires physiotherapy treatment to recover and/or maintain as much functional status as possible after experiencing neurological damage. The neurological physiotherapy treatments provided differed between patients, due in part to the health services available in each location and the patients' family circumstances. Two subthemes emerged: treatment options and family involvement.

#### 3.1.1 Treatment options

The treatment options reported suggest that the neurological treatments received by each patient differed from one another. Some of the patients had only sought treatment from public health services. Several patients had also sought treatment from private services in different cities in Spain, many of them on a temporary basis, and had subsequently discontinued treatment. It is important to note that the patients themselves displayed differences when living in urban areas (they had better access to specialized health services) compared to those living in rural areas.

I was in X for 4 months, then I was in Y and Z doing rehabilitation, and I was getting somewhat better. I returned from Z to X, but I can't remember how long I was there. I was also working with several private physiotherapists. (P‐1). (Each letter represents a different city in Spain)

During my stay in hospital, I already had a physiotherapist assigned to me, but when I was discharged I only had the physiotherapy treatment provided by the Seguridad Social [the Spanish public health system], because I was told that I no longer needed it. I spent about two years receiving physiotherapy several times a week. I've also been going to the swimming pool, but without a physiotherapist, just with the instructor who was there. (P‐3)

I received physiotherapy while I was in hospital, but when I returned to my village, the public health facility there did not accept me, so I continue to do some of the exercises I learned in hospital at home as best I can. (P‐10)

#### 3.1.2 Family involvement

Family involvement was understood as the participation of family members in both treatment and the search for treatments to improve the health status of their relatives with ABI.

My mother also took me to J (a city in Spain). I had an educational psychologist there, a physiotherapist, etc. I've improved a lot since then because at first, I couldn't stand on my own and then I could walk, barely, but I could walk. Before, I couldn't even sit up on my own. (P‐33)

Several patients expressed the need for more education for their relatives in the process of recovery from ABI.

The people around us need help because they're very important in this type of case, and physiotherapists have a lot to offer in this regard. The people around us aren't qualified professionals and it's crucial that physiotherapists train family members to collaborate in our recovery. After all, they're the ones who are with us 24 hours a day. (P‐2, P‐17, P‐19, P‐23, P‐24, P‐26)

### 3.2 Theme 2: Life changes

Patients experienced a number of changes in their lives post‐ABI that continue to have a negative impact. These changes affect their physical condition, their financial situation and their personal lives. Three subthemes emerged: physical changes, financial changes and personal changes.

#### 3.2.1 Physical changes

Physical changes are physical modifications that patients experience post‐ABI that are perceived as functional losses or difficulties: impaired gait, loss of balance, loss of manual dexterity, cognitive impairment, loss of coordination, difficulty performing ADLs and loss of autonomy. A common trend that emerged in most interviews was related to aspects of their physical deterioration (both objective and perceived). Also, those who lived in rural areas felt closer to friends and neighbours.

I feel very impaired. I feel impaired when it comes to doing anything. I have no balance, so I always have to use the walking frame or the wheelchair. (P‐5, P‐10, P‐18)

Yes, I feel impaired because sometimes I can't fasten my buttons because of my terrible fine motor skills, and it's even worse with my left hand. I pick something up and I drop it. I also find it very difficult to spread stuff and fasten my underwear. (P‐6)

In my village I can move around better because my house has no steps, so I can go in and out more freely. It's also a small village, so we all know each other. They're familiar with my situation, so it's not embarrassing for me. (P‐7, P‐11)

#### 3.2.2 Financial changes

Financial changes include any financial changes occurring during the course of the condition. The following were identified: losing jobs, losing career opportunities and losing money.

I felt like a wreck, just like I feel now. Everything my parents had, which was meant for the welfare of all my siblings, went down the drain, through no fault of my own. (P‐5)

I know I won't be able to stand on my own feet again in terms of work, but if I'm lucky enough, I might be able to find a job in a suitable position. It's very difficult around here though. (P‐11)

All the treatment I've received so far has required huge financial sacrifice for my family, but thanks to them, I've been able to get therapy for a while. Otherwise, I wouldn't have received any at all since I was discharged from hospital. Besides, my mother has had to put aside the family business because of me, adding to my family's financial burden. (P‐1)

#### 3.2.3 Personal changes

Personal changes involve negative changes to patients' relational circumstances as a result of their condition, leading to personal losses: loss of a partner, loss of friendships, family losses and personality changes.

All the changes that have taken place in my life have been for the worse. My partner, for example. I had a boyfriend, but when I got sick, I couldn't even manage my own life… I couldn't even stand up… and so I ended up alone. (P‐6)

My relationship with my girlfriend was short‐lived. She couldn't accept the new circumstances. (P‐9)

At the beginning, my friends did come to see me and were concerned about me, but after so long, when they saw that I couldn't keep up with them anymore, I've found that I'm very much alone, with the company of my family and siblings, but few people outside the family. (P‐15)

### 3.3 Theme 3: Feelings about their condition

Patients experience a range of feelings and emotions relating to their ABI and the way they feel within society. These feelings are often negative and relate to how they feel about themselves and how others make them feel. Two subthemes emerged: intrinsic feelings and extrinsic feelings.

#### 3.3.1 Intrinsic feelings

Intrinsic feelings are emotional manifestations arising from patients' personal views about their physical and personal state post‐ABI. Feelings expressed included sadness, hope, desire to cry, depression, guilt, loneliness, hopelessness, denial, fear of the future and low self‐esteem.

I used to think, ‘well, I can't move my left side, but I can get over this with physiotherapy in a week’. That was frustrating, because a week went by, a month went by, another month went by, and I was the same and I felt terrible. At some point, I realised that I wouldn't change. (P‐2)

The way I'm walking at the moment drives me to despair, but I try to control it. That's one thing I've learnt. Do you know what it's like to go out on the street for the first time with one of those walking frames… a tripod, and everyone starts asking you what happened to you? Do you know what that's like? I know there are people who try to hide from it. (P‐10)

I said I don't want to, I won't accept this, I don't want this life, I don't want to be in a wheelchair all my life depending on everybody. I don't think I've accepted it now, but I think I've resigned myself to it. (P‐16)

Yes, I'm quite afraid of the future… I can't walk and my world has fallen apart. But after what happened to me… I'm not afraid anymore. But nobody can take away the feeling of loneliness. (P‐5, P‐31)

#### 3.3.2 Extrinsic feelings

Extrinsic feelings refer to negative emotional manifestations experienced by patients in relation to the way in which society views and treats them. Some of the feelings expressed are feelings of inferiority, feelings of pity, sympathy, lack of support and feelings of being forgotten by others.

When I was younger, other kids made fun of me at school. But the worst time for me was when I came to live in X, because at secondary school they started to bully. I spent a long time keeping it to myself, but one day I couldn't take it anymore and my mother realised and I had to tell her everything. (P‐30)

I feel very bad, because they don't know how it feels, they haven't had an accident, and they don't know what it feels like. Sometimes I've felt belittled and sometimes I haven't… They certainly don't put themselves in your shoes. (P‐17)

### 3.4 Theme 4: Aspirations for the future

Patients voiced a number of wishes and desires for the future that have an impact on their current lives. They varied from patient to patient, but most patients yearned for a personal and professional life like the one they had pre‐ABI.

#### 3.4.1 Personal aspirations

Personal aspirations are wishes or desires expressed by patients about their future personal lives. The personal aspirations expressed include having a partner, having children, making a full recovery, buying a house and returning to their pre‐ABI lives.

I'd like to buy a house, find a girlfriend…. (P‐6)

I'd like to have a driving licence and a woman to love, to be independent…. (P‐1)

#### 3.4.2 Professional or career aspirations

What I really want is to go back to work in the factory and have my little boy. I'm going to go back to work as soon as I get a bit better. (P‐3)

I'd like to be able to help my family in the company and advance in my career. (P‐4)

What I would really like to do is to start working now, but some jobs can be challenging. I think I could work behind a desk or on a computer perfectly well, but I'd have to finish my university education…. (P‐6)

## 4 DISCUSSION

The aim of this study was to gain insight into ABI patients' perceptions of their condition and rehabilitation process so that physiotherapists (and other related rehabilitation professionals) can approach their treatment in a more comprehensive, satisfactory manner.

Physiotherapy is of utmost importance for patients with ABI.^19^ Some of the studies consulted resports that ABI patients receive physiotherapy treatment at their referral hospitals. Despite this, many patients prefer to seek additional, specific physiotherapy treatment (and other therapies that are complementary to their rehabilitation process) at private facilities, due to the long waiting lists at public facilities.^20^

There is a window of enhanced neuroplasticity early after stroke, during which the brain's dynamic response to injury is heightened and rehabilitation might be particularly effective.^21^ Exercise training should be incorporated into the development of neurorehabilitative treatments for long‐term brain injury survivors, and improvements are necessary in all areas of impairment and activity limitations.^21^, ^22^

The Swedish Agency for Health Technology Assessment and Assessment of Social Services has assessed quantitative and qualitative studies on rehabilitation for individuals with TBI through systematic reviews.^23^ As few high‐quality studies were identified, it was not possible to assess the effects of early rehabilitation, rehabilitation for chronic patients, rehabilitation in supported living or specialized rehabilitation for individuals with moderate to severe TBI. What was clear from these studies is that individuals with TBI experienced limited access to rehabilitation services and perceived the interventions that they received as insufficiently coordinated.

Regarding the family sphere, participants were unanimous in highlighting the significance of the family nucleus in their recovery and subsequent return to their ADLs as the only people offering support and understanding of their condition. Other studies report that families require further training to be able to assist in the treatment of their relatives^24^, ^25^, ^26^ and seek treatment options, for which they fully rely on physiotherapists.^27^ Families are key in helping to move the recovery process forward. Sometimes, physiotherapists work as if they were the only people able to help patients, downplaying the importance of the family's involvement in their recovery despite requests from these families to become more involved. The family can improve outcomes over time due to increased and more efficient home practice between sessions.

Another relevant consideration is the perceived changes that ABI has brought about in patients' lives. Several authors corroborate the physical changes identified in our study and characterize them as sequelae resulting from ABI.^28^, ^29^, ^30^, ^31^, ^32^, ^33^, ^34^ Although it is taken for granted that ABI patients may experience significant financial and personal changes, it is sometimes difficult for professionals to understand this process and for patients to accept it.^32^ These changes affect not only the physical and psychological spheres but also the family and financial spheres, and can lead to many material and personal losses resulting in a reduced desire for improvement and recovery. Patients' feelings also change in the face of the illness. Lee^30^ confirms that depression, frustration and failure to accept their new life are the most common feelings among these patients and are primarily due to loss of functional status, as reported in this study. Physiotherapists focus on providing rehabilitation treatment to patients with ABI, but sometimes forget their role as professionals who spend a considerable amount of time with these patients and are instrumental in their recovery. Without realizing it, they ignore patients' feelings and avoid becoming involved with them so as not to become caught up in the difficult process of acceptance. This has received little attention in the literature, unlike patients' aspirations for the future. This study provides professionals with valuable information because it offers insight into ABI patients' perceptions of their condition and recovery process so that physiotherapists can approach their treatment in a more comprehensive and humane manner.

Although this study is novel at the Spanish level, seeks the views of individuals with ABI across a large territory and is gender‐balanced, it has a number of limitations. The mean number of years since the ABI diagnosis was 5.4, meaning that the opinions of individuals with recent or very late onset of ABI could not be included. Our study focused on specific time periods. Therefore, future studies should broaden the range of patients to include the views of newly injured, long‐term and chronically ill patients. Despite efforts to ensure rigorous data triangulation by integrating focus group and individual interview data,^35^ limitations may exist. Another limitation is that a single person performed the qualitative analysis of the data. Although the focus group moderator made an effort to include the views of all participants, not everyone contributed equally to the discussion and, in the telephone interviews, participants were free to express their feelings and perceptions without fear of judgement. Both formats were considered equally in the study, encompassing a wide range of perspectives from a representative sample. However, these results may not be applicable to neighbouring countries, as they do not share a common health system.

Although the life‐course perspective provides a useful conceptual framework for research attending to the diversity and heterogeneity of life‐course trajectories, this strength may also be a challenge, given the increasing complexity of societies and cultures around the world. The findings of our study, which took place in Spain, are consistent with results from other Western countries^36^ suggesting that the life‐course perspective may have broader applicability. Future research is warranted using cross‐cultural applications of this perspective to better understand how adults with ABI are ageing with a disability across the globe. Further research into this issue is required, as ABI patients feel neglected by health professionals. Patients' families should also be included in this type of research to corroborate the information provided in this study, as well as larger numbers of participants. Physiotherapists should become more involved and participate more frequently in research to advance this profession. Moreover, different services involved in rehabilitation could seek to become more integrated and perhaps suggestions could be made regarding the value of further research to build upon this study in the future.

## 5 CONCLUSION

In conclusion, the course of ABI and the process of recovery lead to multiple changes in the lives of patients and their families. In addition, the feelings caused by the condition and/or the society surrounding these patients are not always positive. ABI patients have a series of aspirations that are thwarted by the sequelae of the condition itself and, although a wide range of treatments are available to these patients, it is never enough for them as they seek to return to ‘the way they were before’.

It is also crucial to be able to work with families to help with the treatment and search for solutions, and they are key to the recovery process.

## CONFLICT OF INTEREST

The author declares no conflict of interest.

## Data Availability

Research data are not shared.
